# Drilling Burr Minimization by Changing Drill Geometry

**DOI:** 10.3390/ma13143207

**Published:** 2020-07-18

**Authors:** Emilia Franczyk, Łukasz Ślusarczyk, Wojciech Zębala

**Affiliations:** Production Engineering Institute, Mechanical Faculty, Cracow University of Technology, Al. Jana Pawła II 37, 31-864 Kraków, Poland; slusarczyk@mech.pk.edu.pl (Ł.Ś.); zebala@mech.pk.edu.pl (W.Z.)

**Keywords:** burrs, deburring, titanium alloy, drilling, cutting forces

## Abstract

This article presents an attempt to solve the problem of the formation of burrs and drilling caps in the process of drilling in difficult-to-cut materials, specifically in the titanium alloy Ti-6Al-4V. In order to eliminate these phenomena, a chamfer of specific length and angle was made on FANAR drill’s margin. Taguchi and ANOVA methods were used to plan and analyze the experiment aimed at determining the optimal geometry of the modified drill. Chamfer with a length of 2 mm and an angle of 10° was selected. In the next stage of research, the values of cutting forces and burr heights obtained during drilling with the original and modified drill were compared for three different feed rate values. It turned out that the introduced changes significantly reduced both the axial cutting force (22–23%) and the height of burrs (10–22%) and caused the complete elimination of the presence of drilling caps. Additionally, a positive correlation between the cutting force and the burr size was found.

## 1. Introduction

Rapid development of industry entails the need to use new types of materials and to improve the methods of their processing. Due to their specific properties, titanium alloys, especially Ti-6Al-4V alloy, are becoming more and more popular. Their most important features are good corrosion resistance, biocompatibility and great strength to weight ratio. Due to these properties, they are used, for example, in aviation, chemical, automotive, military and space industries, as well as in medicine (implantology) [1,2]. On the other hand, such materials have a significant disadvantage—their processing causes many difficulties. This is due to their very good mechanical properties, low thermal conductivity and strong chemical reactivity. During cutting, high temperatures, vibrations, friction and high mechanical loads appear on both the workpiece and the tool [1]. Due to the occurrence of high temperatures and friction forces in the machining process, tools wear quickly [3]. Saketi et al. demonstrated that complex physical and chemical mechanisms of tool wear exist while drilling in Ti-6Al-4V, and that they may vary depending on the cutting parameters [4]. In order to ensure the desired quality of items manufactured from such materials, proper selection of process parameters and the use of appropriate tools are required. This also applies to drilling processes. 

One of the problems that arise during such processes, also related to titanium alloys, is the phenomenon of burr formation on workpiece surfaces. The ISO-13715 standard defines a burr as the deviation outside the ideal geometrical shape of an edge. Their formation is associated with the occurrence of plastic deformations present in the area where the tool enters or leaves the workpiece [5,6]. There are a number of studies on phenomena related to burr formation. Abdelhafeez et al. [7] showed that parameters such as feed and cutting speed have a significant impact on the size of burrs in the drilling process. Patil et al. [5] also demonstrated the influence of feed and cutting speed, as well as the tool geometry, and exposed the interactions between these factors. Dornfeld et al. [6] indicated that drill geometry (helix angle, split point vs. helical point, lip relief angle, point angle) has a significant impact on height and thickness of burrs generated during drilling. Bahce et al. [8] showed that the burr height is influenced by the shape of the workpiece output surface. Nithin et al. [9] highlighted the impact of cooling conditions (dry/wet drilling) and drilling aspect ratio. The impact of the type of cooling was also demonstrated by Biermann et al. [10]. Based on the research done in CFRP/titanium/aluminum stacks, Rimpault et al. [11] showed that the size of burrs also depends on the level of tool wear. Joy et al. [3] proved that, depending on the configuration of cutting parameters (cutting speed, feed, temperature), different values of cutting forces are obtained in the process. As noted by Rimpault et al. [11], there is a correlation between the values of these forces and the size of burrs formed. Analytical model of burr formation in Ti-6Al-4V alloy and its experimental verification was already presented in great detail by Rana et al. [12]. Another model (specific for high-speed microdrilling) was developed by Mittal et al. [13].

The presence of burrs on the edges of objects is a very unfavorable phenomenon. It may hinder or completely prevent the subsequent assembly of manufactured elements, cause damage to neighboring parts or lead to personal injury. Abdelhafeez et al. [14] also proved that the presence of burrs on the edges of drilled holes affects the fatigue strength of components made of Ti-6Al-4V alloy. Thus, problems caused by the presence of burrs force manufacturers to take appropriate action. One of the common solutions is the use of additional deburring operations. This is inseparably connected with the extended cycle time, the use of additional tools and the increased amount of labor. As a result, production efficiency decreases while its costs increase. Therefore, it seems that the method of minimizing the phenomenon of burr formation during the drilling process would be a better solution.

Although it is not possible to completely avoid burrs, their size can be minimalized by process optimization. Various methods have been developed in order to control the size of burrs and to remove them without the use of additional operations. The most common method is to optimize the process by appropriate selection of the drilling parameters, the correct choice of the tool material and geometry and the control of its wear. Drilling burr control charts (DBCCs), used to predict and control the size of burrs depending on various factors, presented for example by Min et al. [15] and Kim et al. [16], may also be useful in this context. The possibility of reducing the size of burrs by implementing ultrasonic vibrations in the drilling process was proved by Chang et al. [17]. Mondal et al. [18] also showed that the size of burrs can be reduced by using support element under the workpiece, and by making pre-drilled holes on the tool’s exit side of the processed material. According to Mahdy [19], making preliminary, chamfered holes (pre-drilling and pre-chamfering) may also be a good solution. Rimpault et al. [11] suggested controlling the size of burrs by monitoring the value of the drilling force and torque.

**Figure 1:**
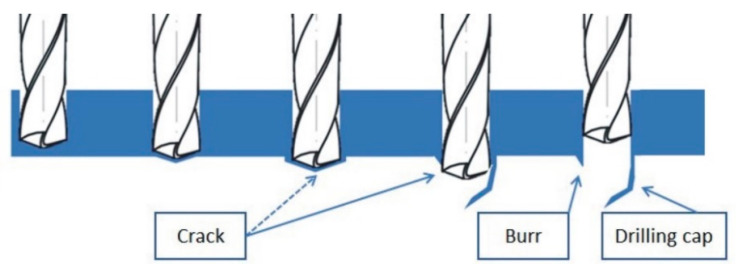
Burr and drilling cap formation scheme.

The drilling process is inseparably associated with occurrence of burrs, and in some cases also with the formation of drilling caps. This phenomenon has been described by several researchers [5,6,20]. It has been shown that the formation of drilling caps is related to the plastic deformations present at the exit edge of the drilled hole. Depending on the degree of ductility of the workpiece, feed and geometry of the tool (especially the point angle), the material may remain unbroken even if some part of the drill has already crossed the exit plane. As the drill advances, stress in the material increases until the occurrence of a fracture. If the loss of material integrity occurs around the tip of the drill, a large, irregular burr appears. However, if the material is torn around the edge of the resulting hole, the burr is smaller, but the drilling cap is formed [5]. The diagram of this process is presented in Figure 1.

Not only the shape and size of the drilling cap depends on the factors mentioned above, but also the burr. As shown in [20], burr width is related to the thickness of the cap. If drilling cap does not fall off during the drilling process, it must be removed in an additional operation. This is particularly problematic in case of intersecting holes or interior cavities [6]. The optimal solution to this problem would be to develop a drilling method that allows making holes with a relatively small burr, while eliminating the phenomenon of drilling caps formation.

Various authors have attempted to develop or modify tools to minimalize burrs or remove them as a part of drilling operations. Kim et al. [21] designed a drill with an additional movable plate which removes burrs on both sides of the drilled element. A similar solution was presented by Yamada et al. [22] and Waschek [23]. Kubota et al. [24] developed a drill with two additional cutting edges, that enables the deburring process in a single machining operation by using contouring control (circular interpolation) in a CNC machining center. Patil et al. [5] obtained smaller burrs when they had chamfered the drill corner and the cutting edge. Ko et al. [20] proposed a new concept of the drill, consisting of changing point angle, flute geometry and length of the chisel edge.

From the above analysis of the literature, it can be concluded that the problem of burr formation is still relevant, and the methods of its minimalization require further development. However various authors have already presented research and simulations related to the formation of burrs in titanium alloys, as well as similar solutions for tool construction [5], this article attempts to optimize the geometry of a specific tool to work with Ti-6Al-4V alloy. Therefore, in order to improve the process of drilling holes in the titanium alloy, modifications were made to the geometry of the Fanar coated solid carbide drill. Conducted research proved that thanks to the introduced modification it was possible to eliminate the formation of drilling caps, and to significantly reduce the size of burrs. The results of this study can provide valuable guidance for cutting tool manufacturers. 

## 2. Materials and Methods

### 2.1. Workpiece Material

A 600 mm × 100 mm flat bar, 10 mm thick, made of Ti-6Al-4V titanium alloy, which composition is shown in Table 1, was used to carry out the research. Physical properties of the alloy are presented in Table 2.

### 2.2. Experimental Setup

**Figure 2:**
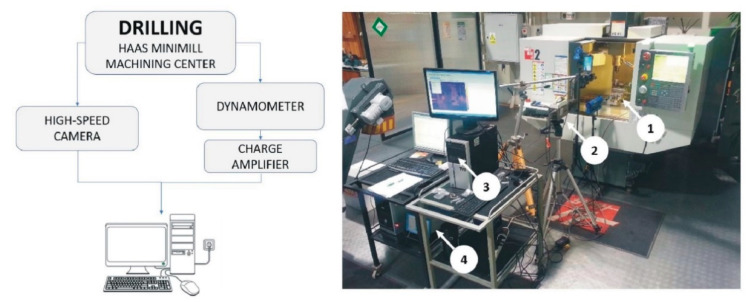
Measuring path scheme (**left**). Research stand (**right**). 1—dynamometer, 2—high-speed camera, 3—PC for data acquisition, 4—charge amplifier.

The test stand consisted of three basic elements: The Haas Minimill CNC machining center, a dynamometer capable of measuring cutting forces and a high-speed camera. A piezoelectric dynamometer Kistler 9257B (Kistler Group, Winterthur, Switzerland) was installed in the working area of the machine, and connected to a PC via Kistler 5070B charge amplifier (Kistler Group, Winterthur, Switzerland). DynoWare software (Version 2825A, Kistler Group, Winterthur, Switzerland) was used to acquire and display data. The test stand configured in this way enabled cutting forces to be measured during the drilling process. The burr formation phenomena were recorded using a Vision Research Phantom v5.2 high-speed camera (Vision Research, Wayne, PA, USA), equipped with a Nikon AF Micro-Nikkor 200 lens (Nikon, Tokyo, Japan) and a Dedocool Cool Light Kit (Mengel Engineering, Virum, Denmark). The scheme of the measuring path and a photograph of the research stand is shown in Figure 2. 

**Figure 3:**
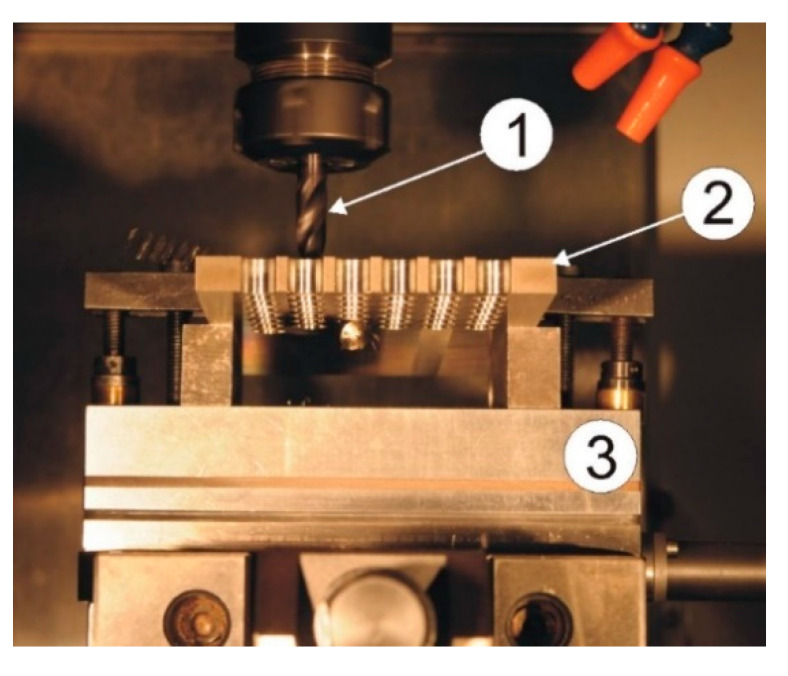
Method of mounting the dynamometer in a machine tool. 1—drill, 2—workpiece, 3—dynamometer.

The dynamometer was attached between the workpiece and the mill table as shown in Figure 3.

### 2.3. Characteristics of Standard and New Concept Drills

**Figure 4:**
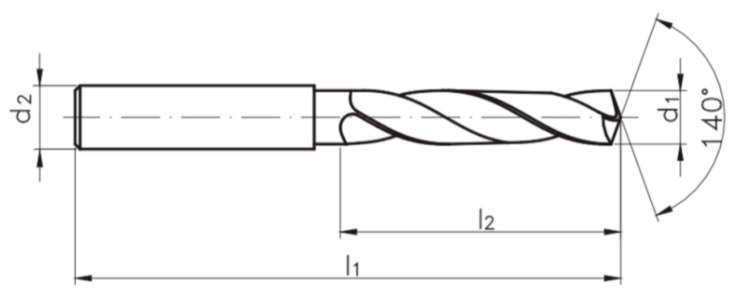
FANAR W9-604013-1000/WK DIN-6537 3xD 10.00 VHM solid carbide twist drill (*d*_1_ = 10 mm, *d*_2_ = 10 mm, *l*_1_ = 89 mm, *l*_2_ = 47 mm).

Initiatory tool for the experiment was the FANAR W9-604013-1000/WK DIN-6537 3xD 10.00 VHM twist drill (Figure 4) (Fanar, Ciechanów, Poland), with a diameter of 10 mm and a point angle of 140°. The tool was made of fine-grained carbide and covered with a titanium aluminum nitride (TiAlN) coating.

**Figure 5:**
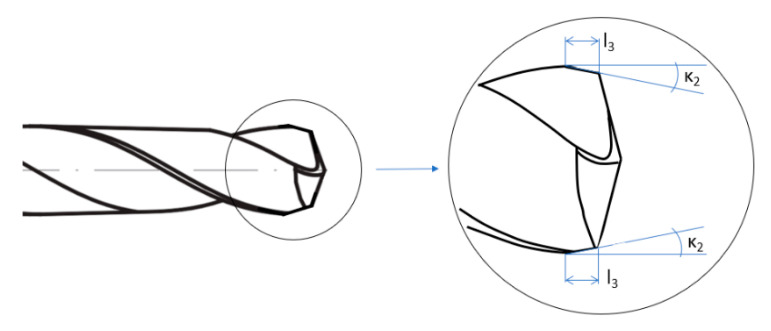
Modified drill.

The modification consisted of making a chamfer of a specific length and angle on the drill margin. Such modified tool is shown in Figure 5. Selected geometrical parameters of the drills are presented in Table 3.

Implementation of the chamfer shortened the main cutting edge from the corner of the drill and simultaneously created an additional cutting edge. This type of modification affected the distribution of cutting forces in the drilling process.

It is assumed that, with a symmetrically loaded drill, the cutting resistance, represented by the resultant cutting force F, is divided into two components, located on the cutting edges: Horizontal force F_c_ and perpendicular to the cutting edge in the vertical plane force F_a_. Next, the force F_a_ can be divided into two components: F_f_ and F_p_. If the force value for one blade is F_f_, it will be 2F_f_ for both blades. Since F_f_ = F_a_sin*κ*_1_, the axial force increases with the increase in the drill point angle (2*κ*).

**Figure 6:**
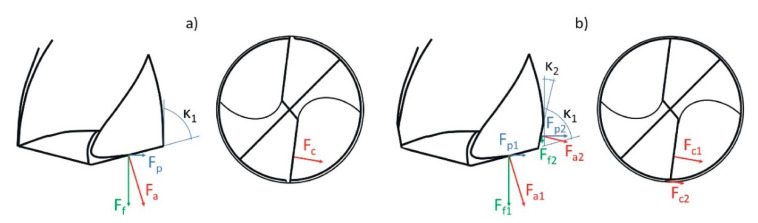
Components of cutting force for a factory (**a**) and modified (**b**) drills.

Creating an additional cutting edge caused a change in the distribution of forces. In this case, the value of the total cutting force F is the sum of the resistances arising on the primary and on the additional cutting edge. Due to great value of the point angle of the additional cutting edge, the axial force F_f2_ is significantly smaller than the axial force F_f1_ that occurs on the original cutting edge. Near the edge of the hole drilled, the amount of force acting in the radial direction is much greater than in the axial direction. The local reduction in the value of the axial force caused that the ejection of the workpiece material, provoked by the tool movement, takes place with less intensity around the edge of the hole than in its center. Thus, a significant reduction in material stress values around the perimeter of the drilled hole was achieved. Distributions of cutting force components for original and modified drills are presented in Figure 6.

### 2.4. Experiment Setup

**Figure 7:**
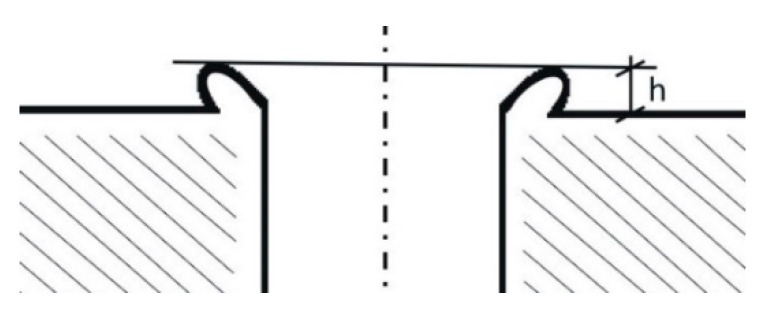
Burr height *h*—value measured during the experiment.

In order to verify the impact of the described modification on the phenomenon of cap formation, and on the size (height) of burrs arising, a number of drilling tests were performed. The study was conducted in a stabilized phase of the process. No increased cutting edge wear was observed, and the modified angles remained stable. The experiment plan, presented in Table 4, was developed using the Taguchi method. Preliminary tests allowed to specify the research scope, i.e., cutting data (feed) and drill geometry. Three factors were tested: feed *f*, length *l*_3_ and chamfer angle *κ*_2_, each on three levels. The measured dependent value was the burr height (Figure 7) and the obtained results were developed using the ANOVA analysis method. Three holes were drilled for each of the tests. In the further part of the work, burr heights resulting from the use of the factory and modified drill were compared for three different feeds. The heights of burrs formed were measured using a profilographometer.

## 3. Results and Analysis

**Figure 8:**
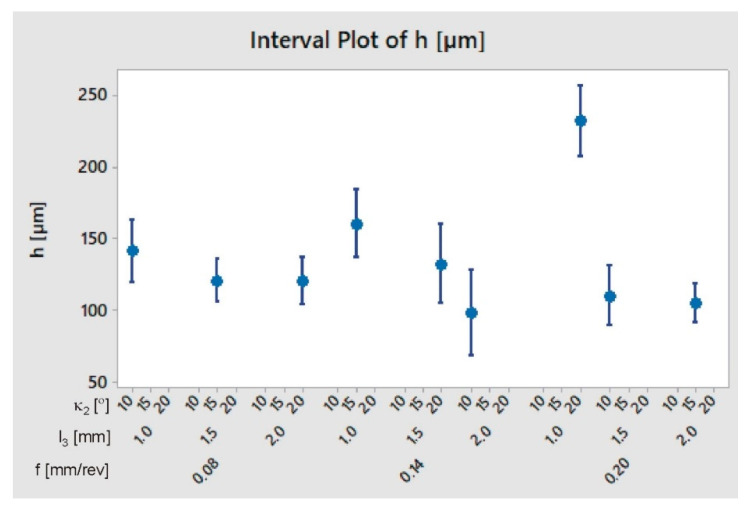
Burr height *h* depending on the shape of the tool used and on the feed.

The results obtained—burr height *h* depending on the shape of the tool used (*l*_3_, *κ*_2_) and the feed f—are summarized in Table 5, and presented in Figure 8.

**Figure 9:**
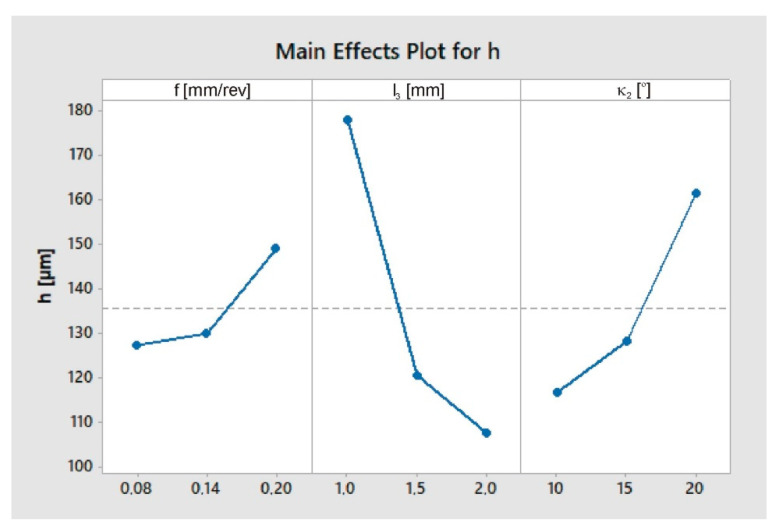
Graphs representing the relation between burr height *h* and the feed *f*, chamfer length *l*_3_ and chamfer angle *κ*_2_.

The ANOVA method was used to process the results. The analysis of variance is shown in Table 6, while the dependence of the burr height *h* on the feed *f*, length *l*_3_ and chamfer angle *κ*_2_ is shown in Figure 9.

**Figure 10:**
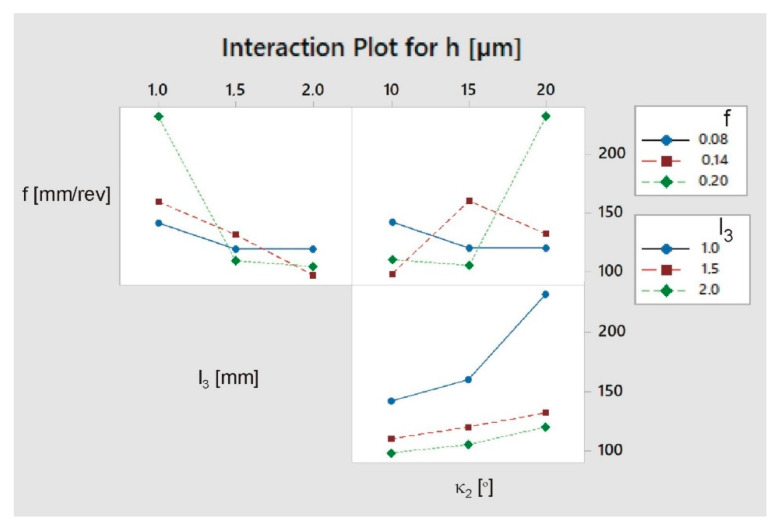
Interaction plot for burr height *h*.

Interactions between the tested factors were also determined. As can be seen in Figure 10, the effect of chamfer length *l*_3_ and angle *κ*_2_ on burr height is greatest for high feed rates. It can also be seen that the effect of the chamfer angle *κ*_2_ is strongest for the shortest chamfer.

As a part of the analysis, the regression equation correlating burr height *h* and the feed *f*, chamfer length *l*_3_ and chamfer angle *κ*_2_ was determined:

(1) $$h=461+2269\cdot f^{2}+88.7\cdot l_{3}{}^{2}+0.427\cdot \kappa_{2}{}^{2}-455\cdot f-336\cdot l_{3}-8.3\cdot \kappa_{2}$$

**Figure 11:**
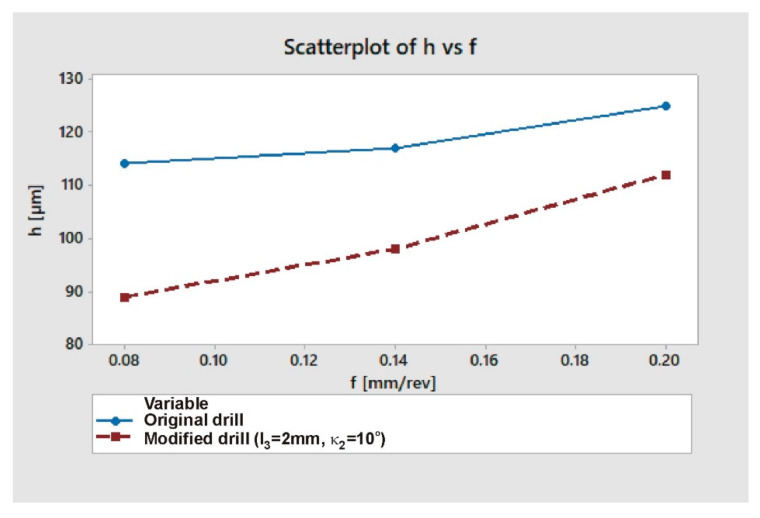
Burr height *h* as a function of feed *f* for factory and modified drills.

Among the tested drill geometries, the version with a chamfer length of *l*_3_ = 2 mm and an angle of *κ*_2_ = 10° proved to be optimal. In the further part of the work, the average values of the burr height for the factory drill and the drill modified in the above manner were compared. Additionally, the axial cutting force (F_f_) values for various feeds were compared between those two drill geometries. The results of these measurements are presented in Table 7. Studies have shown that the change allowed for a significant reduction in the size of burrs (Figure 11). The results obtained are in line with the models presented by Rana et al. [12] and Mittal et al. [13].

It can be seen that the higher the feed, the smaller the relative effect. After applying the drill modification, for *f* = 0.20 mm/rev the burr height was lower by 10%, and for *f* = 0.08 mm/rev by 22%. The relative change in burr size depending on the feed, for extreme values of this parameter (*f* = 0.08–0.20 mm/rev) was 9% for the factory drill, and 20% for the modified drill. 

**Figure 12:**
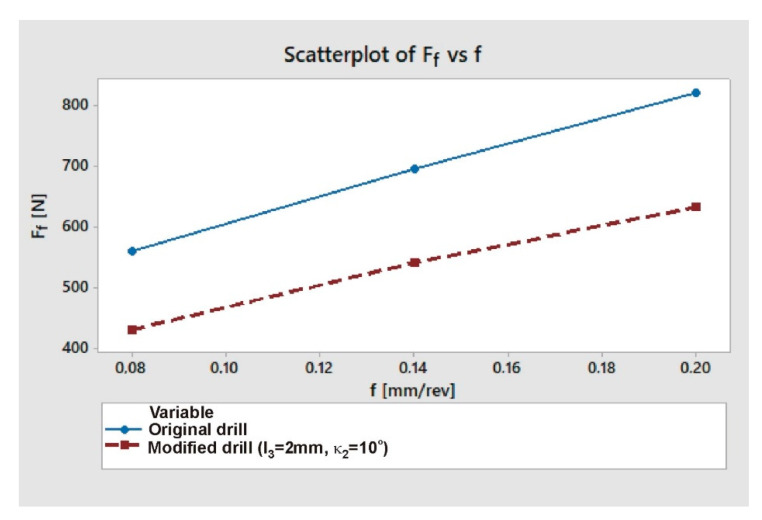
Axial drilling force F_f_ as a function of feed *f* for factory and modified drills.

**Figure 13:**
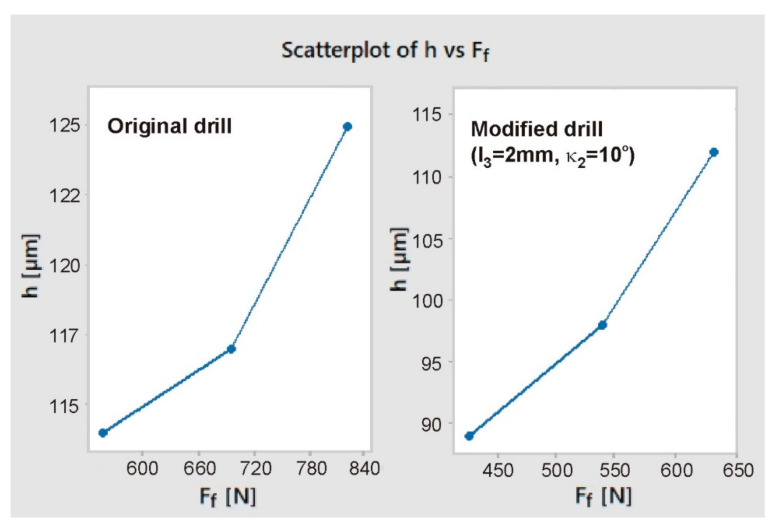
Burr height *h* as a function of axial drilling force F_f_.

As can be seen in Figure 12, for all feed rate values the cutting force was significantly lower when drilling with a modified drill (approx. 22–23%). The data presented in Figure 11 and Figure 12 prove that, as the feed increases, both the cutting force and the burr height increase also. For both drill geometries a positive correlation can be noticed between axial drilling force F_f_ and burr height *h* (Figure 13).

**Figure 14:**
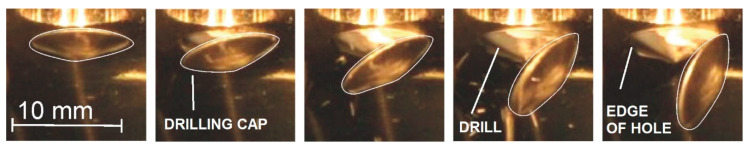
Camera frames showing subsequent stages of drilling with a factory drill.

**Figure 15:**
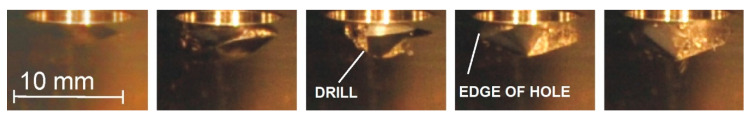
Camera frames showing subsequent stages of drilling with a modified drill.

In each case, the drilling process was recorded using a high-speed camera. When using the factory drill, regardless of the feed used, drilling caps were formed and later they had to be removed from the edge of the hole mechanically. In the case of a modified drill, regardless of the feed value, drilling caps did not appear. Figure 14 and Figure 15 present camera frames from the drilling process with a factory and modified drill, respectively.

## 4. Conclusions

A novelty presented in the paper is the proposed method of burr minimizing by modifying the geometry of the cemented carbide drill working in a titanium alloy. Implemented modifications (chamfer length 2 mm, chamfer angle 10°) resulted in relatively large reduction in the size of burrs, and complete elimination of the phenomenon of drilling cap formation. Depending on the feed value, burr height decreased in a range from 10 to 22%. Thus, the necessity of mechanical removal of caps and the need for additional operations aimed at reducing the size of burrs were eliminated. The change introduced in the drill geometry, which is a technologically simple and low-cost solution, proved to be effective. Used in industry, by reducing the number of tools, operations and energy consumed, it could have a positive effect in terms of reduction of cost and environmental impact.

Studies have shown that the size of burrs depends on the feed value. The drilling process with a modified drill seems to be more sensitive to changes in this parameter. For the original drill, there was a 9% change in the burr height between the extreme values of the tested feed (0.08–0.20 mm/rev). For the modified drill it was 22%, respectively. Based on these results, it can be expected that at higher feeds the effect will decrease, and it even may become negative. It is then possible to further optimize the shape of the drill to increase the effect of minimalizing the size of burrs. The desirable solution is to obtain the greatest effect possible for relatively high feed rates so as to ensure, apart from satisfactory hole edge quality, high machining efficiency.

The cutting forces decreased with a use of modified drill (22–23% depending on the feed value), which can have a positive effect on the tool life. The existence of a correlation between the cutting force and the size of burrs allows, by measuring this force during the drilling process, to estimate the size of burrs in real time. After proper validation, consisting of determining the relationship between burr height and cutting force for a given material, tool and cutting parameters, such solution could become a common method of manufacturing process control.

In the next stage of work it is also advisable to make wear curves for both tools. Comparison of their endurance will allow to estimate the production potential of the modified drill. Given that the cutting forces have decreased after the drill has been modified, one can hope that its service life will be satisfactory, or even better than the original drill.

The limitation for the experiment was the type of tool and material used. The further course of this research should include examining the impact of proposed modification on other types of drills (geometry, material, coating), so as to derive more general conclusions about the effect brought by implementation of a chamfer. Similar tests should also be carried out for other difficult-to-cut materials to check the effect of hardness, adhesion, etc. on the phenomenon of burr formation and on the effectiveness of chamfering operation. Information obtained from such experiments would, in practice, allow for a wider choice of material depending on the application and the acceptable size of burr.

## Figures and Tables

**Table 1:** Chemical composition of Ti-6Al-4V titanium alloy.

Element	Al	V	O	Fe	N	C	H	Ti
**Weight (%)**	6.10	4.13	0.10	0.05	0.01	<0.01	0.003	Balance

**Table 2:** Physical properties of Ti-6Al-4V titanium alloy.

Tensile Strength (MPa)	Yield Strength 0.2% (MPa)	Elongation (%)	Reduction of Area (%)	Hardness (HRC)
1000	900	15	41	30

**Table 3:** Geometrical parameters of the drills.

Point Angle (°)	Chisel Edge Length (mm)	Chisel Edge Angle (°)	Rake Angle (°)	Clearance Angle (°)	Clearance Angle of the Chamfered Part (°)
0.01	0.2	130	30	8	14

**Table 4:** Experiment plan developed according to Taguchi method.

Test No.	1	2	3	4	5	6	7	8	9
***f* [mm/rev]**	0.08	0.08	0.08	0.14	0.14	0.14	0.20	0.20	0.20
***l*_3_ [mm]**	1.0	1.5	2.0	1.0	1.5	2.0	1.0	1.5	2.0
***κ*_2_ [** **°]**	10	15	20	15	20	10	20	10	15

**Table 5:** Burr height depending on the shape of the tool used and on the feed.

Test No.	*f* [mm/rev]	*l*_3_ [mm]	*κ*_2_ [°]	Mean *h* [µm]	σ_h_ [µm]
1	0.08	1.0	10	142	8.7
2	0.08	1.5	15	120	6.0
3	0.08	2.0	20	120	6.5
4	0.14	1.0	15	160	9.5
5	0.14	1.5	20	132	11.1
6	0.14	2.0	10	98	12.0
7	0.20	1.0	20	232	10.0
8	0.20	1.5	10	110	8.5
9	0.20	2.0	15	105	5.5

**Table 6:** Analysis of ANOVA variance for burr height *h*.

Source	DF	Adj SS	Adj MS	F-Value	*p*-Value
***f*[mm/rev]**	2	837.6	418.8	0.86	0.538
***l*** **_3_** **[mm]**	2	8402.9	4201.4	8.63	0.104
***κ*** **_2_ [°]**	2	3220.2	1610.1	3.31	0.232
**Error**	2	973.6	486.8		
**Total**	8	13,434.2			

**Table 7:** Burr height and axial force value depending on the type of drill used.

Drill Type	*f* [mm/rev]	*h* [mm]	F_f_ [N]
Original drill	0.08	114	559
	0.14	117	696
	0.20	125	820
Modified drill	0.08	89	430
	0.14	98	540
	0.20	112	632
